# Lack of complex I is associated with oncocytic thyroid tumours

**DOI:** 10.1038/sj.bjc.6605028

**Published:** 2009-04-07

**Authors:** F A Zimmermann, J A Mayr, D Neureiter, R Feichtinger, B Alinger, N D Jones, W Eder, W Sperl, B Kofler

**Affiliations:** 1 Department of Paediatrics, University Hospital Salzburg, Paracelsus Medical University, Muellner Hauptstrasse 48, A-5020 Salzburg, Austria; 2 Department of Pathology, Paracelsus Medical University, Muellner Hauptstrasse 48, A-5020 Salzburg, Austria

**Keywords:** complex I deficiency, oncocytic thyroid tumour, respiratory chain

## Abstract

Oncocytic tumours are characterised by hyperproliferation of mitochondria. We immunohistochemically analysed all enzymes of the oxidative phosphorylation system in 19 oncocytic thyroid tumours. A specific lack of complex I was detected, which was expressed at <5% of the level determined in surrounding non-cancerous tissue.

Oncocytic tumours and oncocytomas are benign or malignant tumours consisting of oxyphilic cells, characterised histologically by a fine granular eosinophilic cytoplasm and an increase in the number of mitochondria ultrastructurally (Tallini, 1998). Although oncocytic tumours are most frequently found in the thyroid gland, kidney and salivary glands, they have also been reported in various other organs, such as the brain (Gallina *et al*, 2006) and the skin (Jih *et al*, 2004).

An association between mitochondrial DNA (mtDNA) mutations and oncocytic thyroid tumours was reported in several studies (Tallini *et al*, 1994; Maximo *et al*, 2002; Bonora *et al*, 2006). A study on 45 oncocytic thyroid tumours identified potentially pathogenic mutations in subunits of complex I (NADH-ubiquinone oxidoreductase) in 53% of the investigated samples (Gasparre *et al*, 2007). However, the effects of the observed mutations on complex I enzyme activity and protein content have not been shown particularly for the remaining cases in which clear evidence for complex I deficiency could not be shown. In contrast, we identified a loss of complex I enzyme activity and protein in 100% of renal oncocytomas (Mayr *et al*, 2008). We hypothesised that thyroid oncocytomas would exhibit a similar deficiency.

In this study, we investigated oncocytic thyroid tumours by immunohistochemical staining to elucidate changes in the mitochondrial energy metabolism in this tumour.

## Materials and methods

### Patients

Human oncocytic thyroid tumour (*n*=19) and follicular thyroid adenoma (*n*=4) specimens were obtained from the Department of Pathology, Paracelsus Private Medical University, Salzburg. The study was carried out in accordance with the guidelines of the local research ethics committee. Clinical parameters are summarised in Table 1.

### Immunohistochemical staining and analysis

For immunohistochemical staining, the following antibodies were used: mouse monoclonal anti-complex I subunit NDUFS4 (1 : 1000; Abcam, Cambridge, UK), mouse monoclonal anti-complex II subunit 70 kDa (1 : 3000; Mitosciences, Eugene, OR, USA), mouse monoclonal anti-complex III subunit core 2 (1 : 1500; Mitosciences), mouse monoclonal anti-complex IV subunit I (1 : 1000; Mitosciences), mouse monoclonal anti-complex V subunit-*α* (1 : 2000; Mitosciences) and mouse monoclonal anti-porin 31HL (1 : 3000; Mitosciences). All antibodies were diluted in Dako antibody diluent with background reducing components (Dako, Glostrup, Denmark).

Tissue sections (5*μ*m) were deparaffinised by three changes of xylene, rehydrated in three changes of absolute 2-propanol followed by heat-induced epitope retrieval in TE-T buffer (10 mM Tris pH 9.0, 1 mM EDTA, 0.05% Tween 20) for 40 min at 95°C and 20 min at room temperature. Sections were washed in distilled H_2_O and equilibrated with phosphate-buffered saline containing 0.5% Tween 20 (PBS-T pH 7.4). Staining was carried out using the Envision Detection System (Dako) according to the manufacturer's instructions followed by visualisation with diaminobenzidine (DAB) for 10 min. Slides were counterstained with haematoxylin.

To quantify differences in expression levels between tumour tissue and the adjacent normal tissue, a scoring system for the staining intensity (0: no staining; 1: weak staining; 2: moderate staining; 3: strong staining) multiplied by the mean percentage of immunopositive cells per high power fields was used. Quantification was carried out independently by two different persons and mean values are displayed.

### Statistical analysis

For statistical analysis, the Wilcoxon matched-pair signed-rank test was used for equality of distributions. The distribution of complex I and complex V were compared between normal tissue and the corresponding tumour tissue, respectively. The distribution of complex I was also compared to complex V in normal tissue.

### Analysis of mtDNA

Two 10 *μ*m sections were collected and deparaffinised by two changes of xylene and absolute ethanol. After evaporation of ethanol, tissues were treated with 200 *μ*l of PCRK (2 mg ml^−1^ Proteinase K, 75 mM Tris-HCl pH 8.8, 20 mM (NH_4_)_2_SO_4_, 0.01% Tween 20) at 60°C for 24 h followed by 95°C for 10 min. Sequence analysis of the mtDNA was carried out as described previously (Mayr *et al*, 2008).

## Results

### Immunohistochemical staining

**Figure 1:**
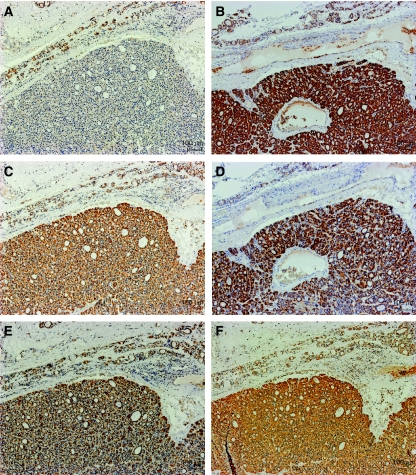
Representative immunohistochemical staining of all respiratory chain enzyme complexes and porin of an oncocytic thyroid adenoma. (**A**) Positive staining for complex I subunit NDUFS4 in normal thyroid tissue (upper part) and immunonegative staining in oncocytic tumour tissue (lower part). (**B**–**E**) Increased expression of respiratory chain complex II subunit 70 kDa (**B**), complex III subunit core 2 (**C**), complex IV subunit I (**D**) and complex V subunit-*α* (**E**) in oncocytic tumour compared with adjacent normal tissue. (**F**) Immunohistochemical staining of porin reveals the characteristic upregulation of mitochondria in oncocytic tumour cells compared with normal thyroid tissue.

Oncocytic thyroid tumour cells were negative or greatly reduced for the complex I subunit, NDUFS4, when compared with the surrounding non-cancerous tissue in all investigated specimens (Figure 1). Immunopositivity for complex II subunit 70 kDa, complex III subunit core 2, complex IV subunit I, complex V subunit-*α* and the mitochondrial membrane protein porin was increased in all tumour samples compared with adjacent normal tissue (Figure 1). These changes were detected in all 17 oncocytic adenomas and the two cases of oncocytic thyroid carcinomas. Follicular thyroid adenomas showed substantial staining for all respiratory chain enzymes and porin (Supplementary Figure 1).

### Statistical analysis

**Figure 2:**
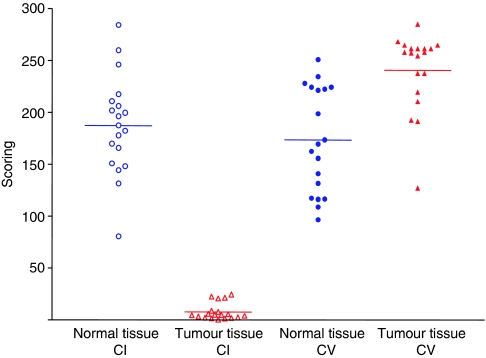
Score values for staining intensity of immunopositive cells in normal and tumour tissues of 19 patients with oncocytic thyroid tumours with complex I and complex V antibodies, respectively.

All investigated oncocytic thyroid tumours showed a significant downregulation of 95% of the complex I subunit NDUFS4, when compared with the adjacent normal tissue (*P*<0.0001; Figure 2). Complex V subunit-*α* showed significant upregulation of 40% (*P*<0.0003) in the tumour tissue (Figure 2). There was no significant difference in normal tissue between complex I and complex V (*P*=1.000). Loss of complex I staining was independent of clinical features, such as endocrine situation, tumour size and histology of the tumour tissues.

Follicular thyroid adenomas showed no statistically significant difference between complex I and complex V staining in the tumour tissues, compared with the adjacent normal tissue (Supplementary Figure 2).

### Mutation analysis of the mitochondrial genome

All seven mitochondrially encoded complex I subunits were sequenced in 18 oncocytic thyroid tumours. Disruptive mutations were found in the complex I genes *ND1*, *ND4*, *ND4L*, *ND5* and *ND6*. Six cases harboured frameshift mutations (3571_3572insC, 10477delT/10952_10953insC, 13235_13236insT, 14339_14340insA, 14603_14604insT), one case of a nonsense mutation (12539G>A) and three cases of mutations causing amino-acid changes in highly conserved domains (3392G>A, 3917A>G and 3946G>A). In eight cases, no potentially pathogenic mutation was found.

## Discussion

Here, we provide strong evidence for an association between specific loss of respiratory chain complex I and oncocytic thyroid tumours. Mutations of mtDNA had previously been identified in oncocytic thyroid tumours; 26.7% of specimens showed disruptive mutations. Additional 26.7% had potentially deleterious missense mutations in one of the seven mitochondrial genes coding for subunits of complex I (Gasparre *et al*, 2007). Similar results were obtained in our samples, where 33% had frameshift mutations, 6% stop mutations and 17% potentially pathogenic point mutations. We cannot exclude mutations in one of the 39 nuclear-encoded subunits. An earlier study has already reported heterozygous mutations in *GRIM-19* (Maximo *et al*, 2005), a subunit of mitochondrial complex I, which is also necessary for assembly. On account of the heterogeneous composition of thyroid tumours, it is difficult to prepare pure tumour homogenates for enzyme measurements or western blot analysis. Gasparre *et al* (2007) were, therefore, not able to report biochemical results of their specimens. It is known from cell culture studies (Hofhaus and Attardi, 1993) and from patients with complex I defects (Ugalde *et al*, 2004a) that severe mutations in different subunits of complex I result in reduced stability or incomplete assembly of the enzyme complex. Moreover, non-mutant subunits of complex I exhibit variable stability (Ugalde *et al*, 2004b). For example, the NDUFS4 subunit is particularly unstable and its stability depends on the presence of the preassembled complex I. Because of this instability of unassembled NDUFS4, we used immunohistochemical staining with an NDUFS4 antibody as a measure of complex I content.

Complex I is an integral component of different apoptotic pathways (Ricci *et al*, 2004; Martinvalet *et al*, 2008). Thus, deficiency of complex I could play a role in tumour formation by interrupting apoptotic pathways. Besides its function as part of the respiratory chain, complex I can be transformed to a potent enzyme of reactive oxygen species (ROS) formation. It was shown that the NDUFS1 subunit of complex I can be proteolytically cleaved by caspase-3, which results in a truncated enzyme complex disposed for ROS production (Ricci *et al*, 2004). In a second, caspase-independent apoptotic pathway granzyme A specifically cleaves subunit NDUFS3 of complex I. This again results in increased generation of ROS, disruption of the mitochondrial transmembrane potential and cell death (Martinvalet *et al*, 2008). Lack of complex I, as identified in oncocytic thyroid tumours, may, therefore, prevent tumour cells from undergoing apoptosis through these pathways.

The clinical parameters shown in Table 1 are typical for the diagnosis oncocytoma and the complex I deficiency was independent of age, gender, tumour size, histology and endocrine situation. All investigated thyroid oncocytomas showed complex I deficiency and most had a benign clinical course; however, 2 of the 19 tumours in this study were carcinomas. Renal oncocytoma, another type of oncocytic tumour with a benign course, was recently found to be associated with complex I deficiency (Gasparre *et al*, 2008; Mayr *et al*, 2008). The oncocytic tumour cell line XTC.UC1, which is derived from a metastasis of a Hürthle cell carcinoma, shows both an insertion mutation in the gene encoding ND1 subunit of complex I and a reduction in complex I activity (Bonora *et al*, 2006). Ishikawa *et al* (2008) reported complex I deficiency caused by mutations in the ND6 gene of complex I in non-oncocytic mouse lung tumours. Interestingly, these mutations increased the metastatic potential of the tumours (Ishikawa *et al*, 2008), whereas complex I deficiency in oxyphilic tumours does not seem to be associated with metastasis. For that reason, complex I deficiency is not considered as a specific feature of benign oncocytic tumours.

To establish that isolated complex I deficiency is a characteristic of oncocytic thyroid tumours, but not a general feature of thyroid tumours, we analysed four follicular thyroid adenoma samples. In contrary to oncocytomas, follicular thyroid adenomas displayed no significant reduction of respiratory chain enzymes.

Loss of complex I was found in all investigated oncocytic thyroid tumour samples. Because of the strict dependence of such tumours on glycolytic metabolism, this unique feature could enable the development of novel targeted therapies for this disease.

## Figures and Tables

**Table 1:** Clinical parameters of the patients with oncocytic thyroid tumours

**Parameter**	**Determined**	**Values: mean (range)**
Age	19/19	55.6 years (27.6–83.8)
*Gender*	19/19	
Male	3/19	
Female	16/19	
*Diagnosis*	19/19	
Adenoma	17/19	
Carcinoma	2/19	
Tumour size	19/19	2.6 cm (1–5.3)
*Endocrine situation*	18/19	
Hypothyroid	1/18	
Euthyroid	14/18	
Hyperthyroid	3/18	
*Goitre*	15/19	
Yes	15/15	
No	0/15	
*Immune thyroiditis*	16/19	
Yes	4/16	
No	12/16	
