# Supplementary figures and images for: Lack of complex I is associated with oncocytic thyroid tumours
Source: Br J Cancer. 2009 Apr 7;100(9):1434–7. doi: 10.1038/sj.bjc.6605028 (PMC2694433; doi:10.1038/sj.bjc.6605028)

## Slide 1
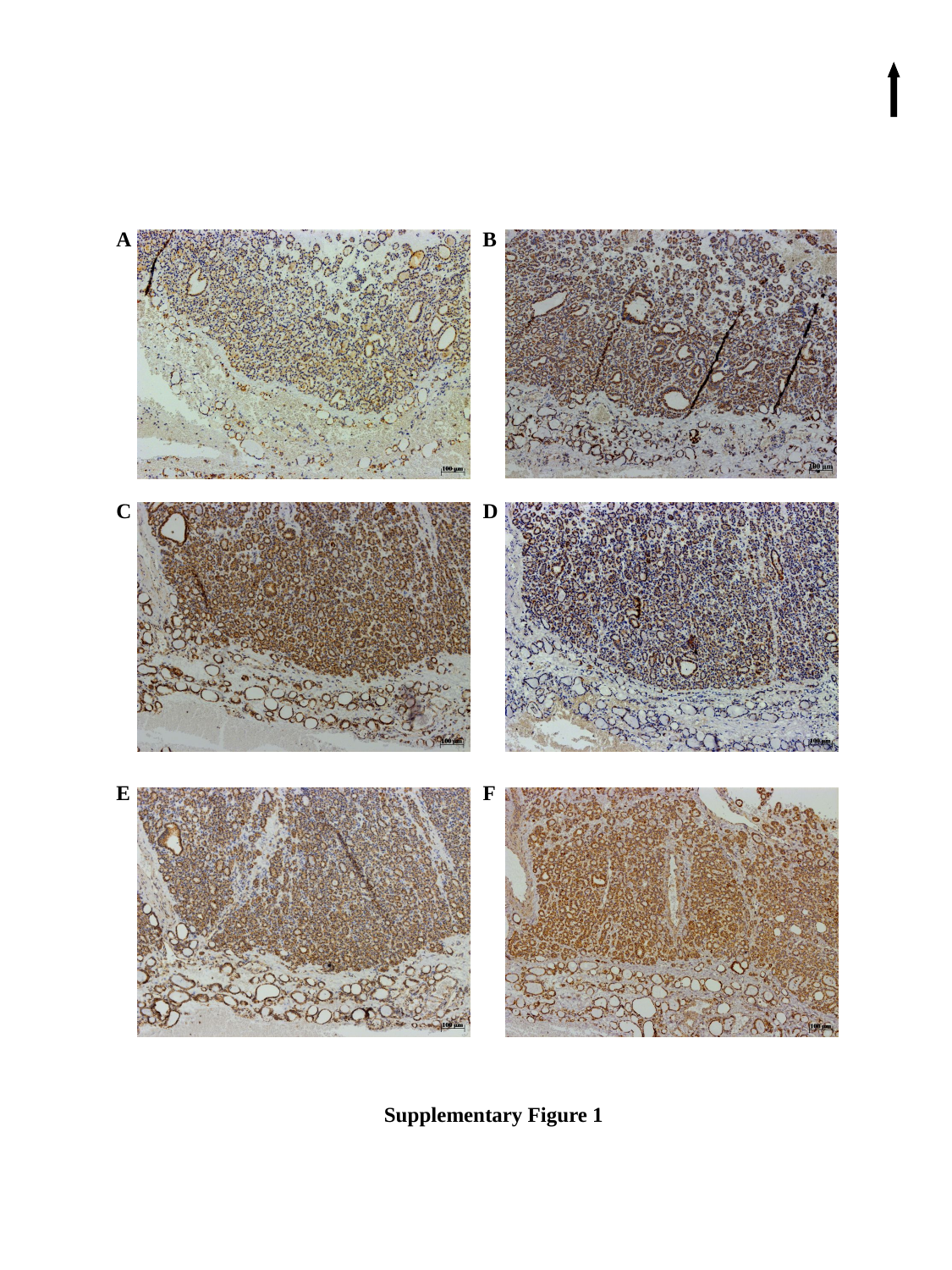

A
B
C
D
E
F
Supplementary Figure 1

Supplement: Supplementary Figure 1 [file 6605028x1.ppt]
