# Supplementary material for: Lack of complex I is associated with oncocytic thyroid tumours
Source: Br J Cancer. 2009 Apr 7;100(9):1434–7. doi: 10.1038/sj.bjc.6605028 (PMC2694433; doi:10.1038/sj.bjc.6605028)
Supplement: Supplementary Figure 2 [file 6605028x2.ppt]

## Slide 1
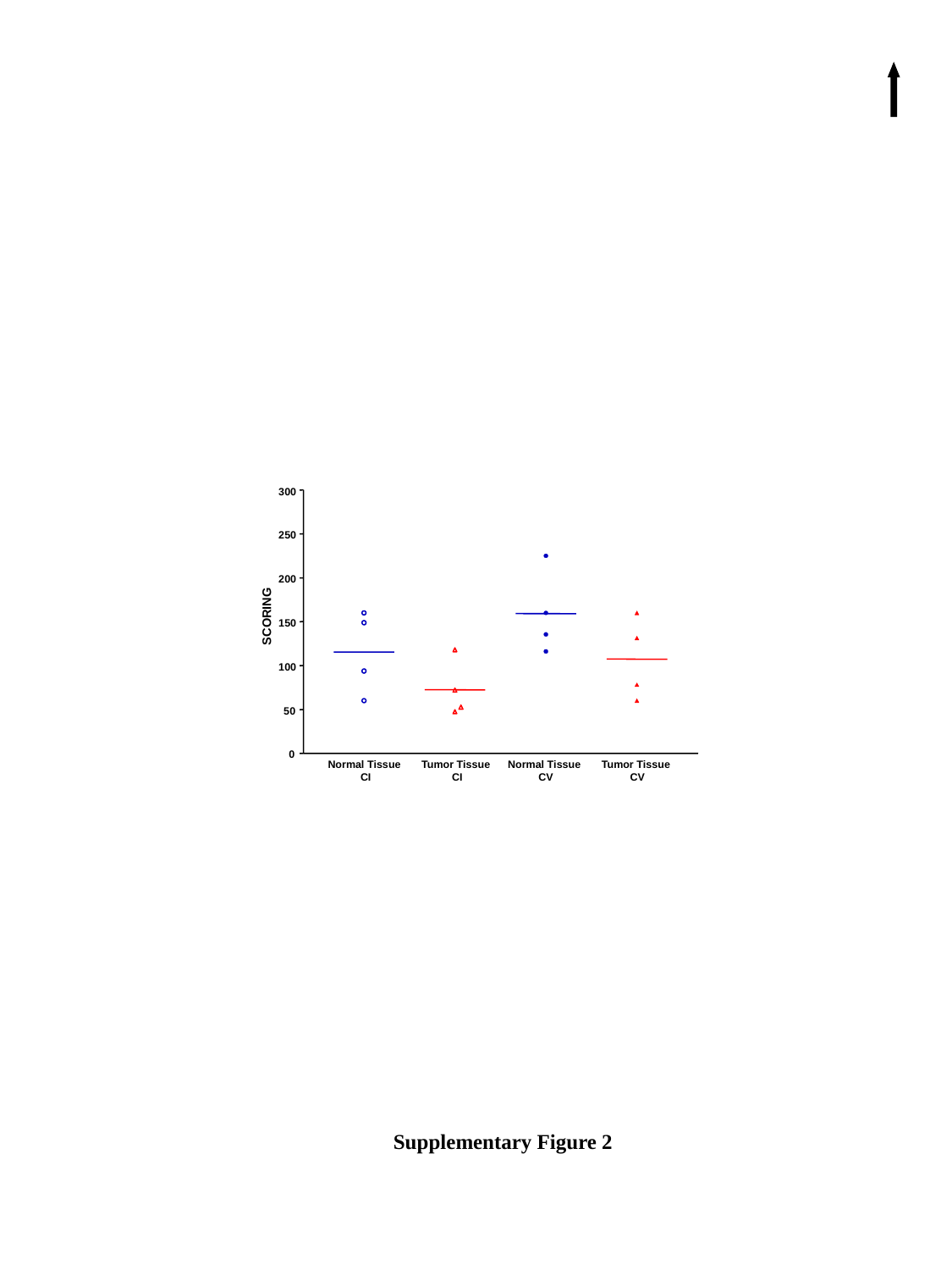

300
250
200
SCORING
150
100
50
0
Normal Tissue
CI
Tumor Tissue
CI
Normal Tissue
CV
Tumor Tissue
CV
Supplementary Figure 2
