# Supplementary material for: Lack of complex I is associated with oncocytic thyroid tumours
Source: Br J Cancer. 2009 Apr 7;100(9):1434–7. doi: 10.1038/sj.bjc.6605028 (PMC2694433; doi:10.1038/sj.bjc.6605028)
Supplement: Supplementary Figure Legends [file 6605028x3.doc]

**Supplementary Figure 1** Immunohistochemical staining of respiratory chain enzymes and porin of a representative follicular thyroid adenoma. (**A-E**) Positive staining of complex I subunit NDUFS4 (A), complex II subunit 70 kDa (B), complex III subunit core 2 (C), complex IV subunit I (D) and complex V subunit alpha (E) in the follicular adenoma (upper part) and the normal thyroid tissue (lower part). (**F**) In contrast to oncocytic adenomas, immunohistochemical staining of porin reveals no upregulation of mitochondria.

**Supplementary Figure 2** Score values for staining-intensity of immunopositive cells in normal and tumour tissue of four patients with follicular thyroid adenomas with complex I and complex V antibodies respectively.
